# Deposit pickers in the Nordic: The role of deposit-refund systems for waste pickers in Stockholm

**DOI:** 10.1177/0734242X241297574

**Published:** 2024-11-28

**Authors:** Nils Johansson

**Affiliations:** Department of Sustainable Development, Environmental Science and Engineering, KTH Royal Institute of Technology, Stockholm, Sweden

**Keywords:** Waste picker, deposit-refund system, informal recycling, scavenging, stigmatization, waste management

## Abstract

This article examines a specific subtype of informal waste picking: deposit picking. Despite its global prevalence, waste picking has neither been extensively studied in the Nordic countries nor in the context of a deposit-refund system. Through interviews and text analyses of waste pickers in Stockholm, Sweden, similarities and differences between deposit picking and traditional waste picking are uncovered. For example, unlike other waste materials, the income from deposits is stable. The focus on beverage containers and the ability of reverse vending machines to sort the containers, lowers the knowledge threshold to begin the activity. The lightweight nature of beverage containers makes collection mobile, and deposit pickers often carry only a bag. The deposit pickers are mainly older, poor and male. Similar to traditional waste pickers, deposit pickers are central to the formal waste system, but their work is invisible, and foreign deposit pickers, in particular, are stigmatized. The dual invisibility of their labour and contributions, coupled with their independence from formal social systems, highlights the need for internal organization and representation within the formal systems.

## Introduction

Waste pickers play a crucial role in waste management around the world. In the economically developing countries, approximately 0.5%–2% of the population make a living by collecting, sorting and selling waste (International Labour Organization, 2018; Medina, 2007; Scheinberg et al., 2016). In many cities like New Delhi and Cairo, the informal sector provides the main collection and sorting services for waste (Wilson et al., 2009, 2010). When informal and formal systems operate in symbiosis, the informal sector demonstrates higher efficiency and recycling rates than the formal systems (Ramusch and Lange, 2013; Wilson et al., 2009). Thus, informal waste pickers significantly contribute to social, environmental and economic benefits in the Global South, as shown in Table 1.

**Table 1.** Reported environmental, economic and social benefits of informal waste picking.

Environment	Economic	Social
Reduces littering Reduces air, soil and water pollution Diverts waste from landfills Conserves natural resources Energy savings Climate change mitigation Preserves the aesthetic values of nature	Reduces waste management and sanitation costs Government funds freed up for other purposes Preserves economic value of resources Job creation reduces incentives for crime Provides pathways to formal systems Facilitates the achievement of policy objectives Generates incomes and an entrepreneurial spirit among marginalized groups Provide secondary resources to the industry	Job creation Poverty reduction Prevents spread of diseases Raises awareness of the material and environmental responsibility Fosters collaborations and social capital Builds community and inclusion Improved mental health through outdoor activities Empowerment

References: Chaturvedi (2009); Chikarmane and Narayan (2009); Gutberlet et al. (2017); Linzner and Lange (2013); Medina (2009); Nzeadibe (2013); Scheinberg et al. (2016); Velis (2017); and Wilson et al. (2009, 2010).

Although waste pickers are relatively well studied, and in some places even integrated and recognized in the Global South, their conditions in the Global North are less acknowledged (Porras Bulla et al., 2021; Scheinberg et al., 2016). In the Global North, informal waste management is primarily seen as a historical phenomenon, which at the turn of the last century was as common in the Global North as in the South (Medina, 2001). The modernization and centralization of waste systems along the transformation of waste into commodities during the 20th century, made it harder for the informal sector to operate in the Global North (Barles, 2014; Rathje and Murphy, 1992). However, research on informal waste management in the Global North has revealed cracks in the formalized systems (Gutberlet et al., 2009; Medina, 2001; Scheinberg et al., 2016). As more people are excluded from societal safety nets while waste generation increases, waste picking has become more prominent even in the affluent parts of the world (Williams, 2013; Wittmer and Parizeau, 2016). Those of us living and operating in the economically affluent part of the world find it hard to ignore the ‘large and growing army of informal recyclers’ (Gowan, 1997: 160).

Although the political act of dumpster diving, especially for food, is relatively well studied in the Global North (Ferrell, 2006; Rombach and Bitsch, 2015), the collection and utilization of other types of waste are considerably less studied. The few studies of waste picking conducted in the Global North have primarily focused on North America (Gowan, 1997; Gutberlet et al., 2009; Heidelberg and Surak, 2020; Medina, 2001; Parizeau, 2017; Porter, 2015) and Central and Southern Europe (Climent Sanjuán and Porras Bulla, 2018; Ramusch et al., 2015; Rendon et al., 2021; Rosa and Cirelli, 2018; Scheinberg et al., 2016). Studying waste picking in the Nordic region not only sheds light on a previously neglected area of waste picking, but opens also up the possibility to examine waste picking in relation to deposit-refund systems (DRSs), where customers pay an extra deposit when purchasing a product, which is refunded at the return.

DRSs have been present in previous studies of waste picking (Ashenmiller, 2009; Parizeau, 2017; Wittmer and Parizeau, 2016), but have rarely been the framework of focus. Therefore, knowledge is lacking on the conditions, opportunities and challenges for waste pickers in a DRS. To improve our understanding of waste pickers’ role and relationship with DRSs are important because these systems are expected to spread as recycling requirements increase (European Commission, 2022). Since it is not the customer who needs to reclaim the deposit, the DRS has been suggested as a policy instrument that internalizes and compensates waste pickers for their labour (Dias et al., 2022; Porras Bulla et al., 2021).

The purpose of this study is to increase the knowledge about waste pickers’ relationships, challenges, opportunities and conditions in a DRS. This will be done by addressing fundamental questions about who, what, where, why, how, and when waste pickers operate, and how their activities are perceived by their surroundings. Data were collected through interviews with, what we refers to as, *deposit pickers* in Stockholm, Sweden and those responsible for the waste systems, as well as supplementary text analyses.

## Background: The dynamics of waste picking

Waste pickers seek, collect, sort, select, extract, process, transport and sell discarded materials and items, partially within or outside formal waste management systems. The space for waste pickers typically arises from institutional deficiencies where waste is not properly collected or sorted (Ezeah & Roberts, 2012) and an increasing demand for waste as a commodity.

### How?

Waste is collected from various sources including households, businesses, streets, bins and landfills (Velis et al., 2012). The tools and modes of transportation depends on how and where waste is picked. For instance, in Gaza, cars and horses are predominantly used to collect and transport waste from street bins (Al-Khatib et al., 2020), whereas in North America, shopping carts are primarily used (Gutberlet et al., 2009). The physically demanding nature of waste picking leads to a higher rate of injuries and health issues compared to other occupations (Gutberlet and Baeder, 2008; Wittmer and Parizeau, 2016), notably cuts and physical soreness (Zolnikov et al., 2021). Many waste pickers, particularly in the South are organized through cooperatives or unions (Buch et al., 2021; Medina, 2000; Zapata Campos et al., 2023), providing them with support, structure, camaraderie, recognition, representation and practical assistance with authorities, compensation, waste storage, tool provision, uniforms and identity cards (Da Silva et al., 2021; Gutberlet and Baeder, 2008; Kain et al., 2022; Velis et al., 2012).

### Why?

In the Global South, organized waste picking, through cooperatives, is viewed primarily as a profession (Medina, 2008). In contrast, in the Global North, the picking activity is primarily seen as a necessary sideline for survival (Rendon, 2020). For example, 97% of waste pickers in Gaza had no other job or means of support (Al-Khatib et al., 2020), and 65% of waste pickers in Brazil considered themselves better off as waste pickers (Ramos et al., 2013). In the Global North, on the other hand, waste picking is often combined with other livelihood activities, such as additional work in agriculture, begging (Rosa and Cirelli, 2018) or as a supplement to social assistance payments (Ashenmiller, 2009; Gutberlet et al., 2009). The primary driving force behind all waste picking activities is to earn money (Medina, 1998). Incomes vary between different locations, ranging from an average of €16 per day and person in Vancouver (Wittmer and Parizeau, 2018), €6 in California (Ashenmiller, 2009) to €1 in New Delhi (Hayami et al., 2006).

### What?

The focus of waste pickers is waste that yields income (Minter, 2015) and can be delivered to a local waste buyer (Al-Khatib et al., 2020). Primarily, not only metals, plastics, paper and glass are picked (Lima et al., 2022; Nzeadibe, 2009), but also electronics, cardboard, newspapers, textiles, rubber and wood (Schenck and Blaauw, 2011). It is almost easier to list what pickers do not embrace. One of the least desired materials is bone residues (Suttibak and Nitivattananon, 2008). Waste pickers also seek reusable items such as food, clothing and furniture for sale or personal use (Ojeda-Benitez et al., 2002; Medina, 2001; Rosa and Cirelli, 2018).

### Who?

Waste pickers belong to the poorest segment of the population in each country (Al-Khatib et al., 2020; Asim et al., 2012; Ezeah et al., 2013; Gutberlet et al., 2009; Kaza et al., 2018; Samson, 2020) and are typically excluded from the formal job market (Wilson et al., 2006). In the Global South, the majority of waste pickers are younger than 30 years (Al-Khatib et al., 2020; Almeida et al., 2017), with all available demographics participating: men, women, the elderly and even children (Adamo, 2014; Adeyemi et al., 2001; Chen et al., 2018; Hunt, 1996; Singh et al., 2023; Wittmer, 2023). The participation of children in waste picking is often due to lack of resources for schooling, forcing them to accompany their parents in their activities (Adamo, 2014; Hunt, 1996).

In the Global North, on the other hand, waste pickers are generally older than 30 (Gowan, 1997; Gutberlet et al., 2009; Tremblay et al., 2010) and predominantly male (Gowan, 1997; Rendon et al., 2021). In Europe, waste pickers can be divided into three categories according to Scheinberg et al. (2016): (i) ethnic minorities such as Roma; (ii) undocumented migrants and (iii) individuals excluded from the job market, such as the homeless.

### When?

Collecting waste from the streets and bins is typically a routine-based activity performed daily, sometimes multiple times a day (Rendon, 2020). Street pickers adjust their routines according to the schedules of waste collectors, ensuring they collect valuable items before they are possessed by the formal system (Lane, 2011; Rosa and Cirelli, 2018). Picking waste is an activity that demands many hours per person and day, averaging, for example, 10 hours in Lahore, Pakistan (Asim et al., 2012) and 5 hours in Victoria, Canada (Gutberlet et al., 2009).

### Where?

Each street picker typically operates within a territory they rhythmically traverse, covering an area of approximately 10–15 km (Asim et al., 2012). Crossing territorial boundaries may lead to conflicts with competing pickers (Gutberlet and Uddin, 2017). Valuable waste is also picked from the ground as litter in places frequented by many people, such as parks and concerts, but also directly from businesses and households (Parizeau, 2017). In exchange, pickers offer services to businesses, such as cleaning or surveillance (Sholanke and Gutberlet, 2022). After collection, waste is sold to middlemen, as industries rarely accept smaller quantities directly from pickers (Ezeah et al., 2013).

### Relationships?

Waste pickers are often stigmatized, humiliated and devalued (Gutberlet, 2010; Porras Bulla et al., 2021; Reno, 2009). Waste picking is typically more contested in the North than in the South (Medina, 2000). This is because the function of waste pickers is more pronounced in areas where authorities lack the capacity to manage waste. In the Global South, there are also many examples of how informal networks have been successfully integrated in formal waste management systems (Aparcana, 2017; Majeed et al., 2017; Samson, 2020; Wilson et al., 2006). In the Global North, on the other hand, waste pickers easily become a disruption to what is regarded as well-functioning waste systems (Scheinberg et al., 2016). Therefore, waste picking has been criminalized in many cities in the Global North, and trash bins have been locked (Parizeau and Lepawsky, 2015; Scheinberg et al., 2016). However, there are also examples of local authorities trying to facilitate waste picking. For example, in Barcelona, the organization of waste pickers is encouraged (Porras Bulla et al., 2021), and in Vancouver, waste pickers are offered specially customized carts (Table 2) (Sholanke and Gutberlet, 2022).

**Table 2.** Reported environmental, economic and social problems of informal waste picking. These problems, however, should be understood contextually. For example, the design of infrastructure such as trash bins largely determines whether and how street pickers may litter.

Environment	Economic	Social
Spread litter and clutter the surroundings Poor handling of hazardous waste, such as e-waste, may release toxins to the environmentIf vehicles are used for picking, energy is consumed with emissions Leaving behind the non-economical waste	Displace valuable materials from formal waste management Loss of job opportunities in the formal sector Waste pickers are at the bottom of the hierarchy and receive inadequate compensation Informal handling increases the risk of illegal waste trading and handling Waste is made inaccessible by locking bins	Health issues such as wounds and infectious diseases Stigmatizing and discriminatory High competition for the most valuable waste and locations can lead to internal conflicts Child labour has been reported in the global south Inequality. The public urban environment is unsafe for women Absence of labour rights Perceived to create insecurity in affluent areas

References: Al-Khatib et al. (2020); Ezeah et al. (2013); Gowan (1997); Hunt (1996); Parizeau and Lepawsky (2015); Porras Bulla et al. (2021); Uddin and Gutberlet (2018); and Wittmer and Parizeau (2018); Zolnikov et al. (2021).

## Methodology

Information for this study was collected through interviews with waste pickers and service providers in Stockholm, Sweden and supplemented with textual analysis.

### Case study

Stockholm, the capital of Sweden, stands out due to its attractiveness, growth, population density, homelessness rates, segregation and the proportion of foreign-born residents. These factors mean that the answers to the fundamental questions about deposit pickers might differ if the study were conducted in the rural areas of Sweden. However, in terms of population dynamics and relations with the waste system, Stockholm shares more similarities with other urban areas in the Nordic region.

Beverages, except dairy products, sold in metal or plastic containers are part of a nationwide DRS in the Nordic countries (Rehnberg, 2010; Swedish Parliament, 2022). This means that consumers pay an extra fee when purchasing a beverage container, which is refunded upon return (Lu et al., 2022). Typically, these containers are returned to a reverse vending machine, which separates them by material and compacts them for transport to a central recycling facility. The DRS is operated by *Returpack*, an organization jointly owned by the Swedish breweries and trade associations. The Swedish DRS was initially established for glass bottles in 1886, expanded to include metal cans in 1981, and plastic bottles in 1992 (Johansson, 2022).

**Figure 1.**
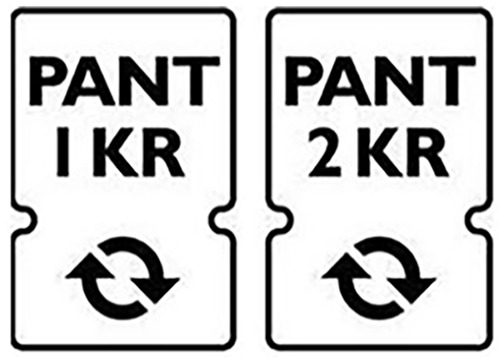
The symbols indicating that the packaging is subject to a deposit for recycling purposes.

The deposit per container varies depending on size, either 1 or 2 Swedish Krona, Figure 1, equivalent to 0.1 or 0.2€. According to the policy objective of Sweden, 90% of the sold containers within the DRS shall be recycled. In 2023, 2.7 billion cans and bottles were returned in Sweden, with 186 million from Stockholm, representing 88.5% of the sold containers (Returpack, 2024). The DRS is one of the most popular environmental policies in Sweden, with significant annual investments in promotional activities. For example, every year, a new version of the ‘Redeem more’ [Swedish: Pantamera] jingle is launched through collaboration with a well-known music artist. While 97% of Sweden’s population reports participating in the system, it is rarely the same person who buys the container that returns it (Du Rietz, 2023).

### Data collection and analysis

Information was gathered through semi-structured interviews (Aldridge, 2014) with waste pickers and service providers. The interviews with waste pickers aimed to learn from their experiences and perspectives by asking basic questions about their background, what, where, why, how and when they pick, and how their picking relates to surrounding actors. See Appendix A for the interview guide.

Respondents were selected following observations of individuals seen searching in bins. This took place in various parts of Stockholm’s city centre. Interviews were conducted during mornings and afternoons. Since waste pickers are a vulnerable respondent group, it was clarified during the request for participation that they could decline the interview without any consequences. In total, 30 waste pickers were approached to participate in the study, 21 of them agreed to participate, see Appendix B for presentation, whereas 9 of them declined to be interviewed. Anonymity was ensured during participation, and no photographs were taken.

Semi-structured interviews were also conducted with service providers, such as those who own and manage municipal waste bins, responsible for the DRS, and supermarkets with reverse vending machines. Questions for service providers focused on their experiences with waste pickers, advantages and disadvantages, and how these are addressed within their operations (Appendix A).

As a complement to the interviews, textual analyses (Drisko and Maschi, 2016) of official documents and news articles were conducted. Articles and documents were searched between the years 2013 and 2023 using ‘Mediearkivet’ database, which collects Swedish official documents and news articles. Keywords used were, if translated to English, ‘deposit’, ‘can’ or ‘bottle’ + ‘picker’ or ‘collector’. Texts were selected that could verify and complement the deposit pickers’ own information and provide an understanding of their situation, relation to the rest of society, legal status and how they are described in the media.

After transcribing the interviews, all collected material was analysed through predetermined themes (Saldaña, 2013) related to the aforementioned basic questions: who, when, where, how and why. The analysis concluded by examining if and how the different themes related to each other.

## Results and discussions

The respondents are presented in Appendix B, specifying their age and country of birth. Below follows an analysis and discussion of their responses.

### Who?

Among the interviewed individuals, the average age was 54 years, with the majority being men (18 out of 21), which is consistent with other waste pickers in the Global North (Gowan, 1997; Gutberlet et al., 2009; Rendon et al., 2021). However, when divided into three different groups in line with Scheinberg et al. (2016), it is clear that age varies depending on origin. The average age for those born in Sweden, 9 out of 21, was 60 years. Ten out of 21 respondents originated from Eastern Europe, primarily Romania, with an average age of 46 years. A smaller proportion, 2 out of 21 individuals, were born in Africa, with a significantly lower average age of 33 years.

Several of those born outside of Sweden stated that housing was a problem, and some of them were homeless. For those born in Sweden, housing was less of an issue; three of them (Person 1, Person 8 and Person 13) stated that they had their own accommodation.

### What?

All pickers stated that they focused only on beverage containers labelled with the deposit symbol (Figure 1). This distinguishes Swedish waste pickers from those in regions outside the Nordic. Traditional waste pickers, particularly in the Global South, target a range of waste fractions for their material value (Lima et al., 2022; Minter, 2015; Ojeda-Benitez et al., 2002; Schenck and Blaauw, 2011).

In other countries with DRSs, such as Canada, waste pickers do not only focus on deposit items but also collect e-waste, food and clothing (Gutberlet et al., 2009; Sholanke and Gutberlet, 2022; Wittmer and Parizeau, 2016). Swedish waste pickers focus on aluminium cans and PET-bottles. One of the pickers stated that he also looked for glass bottles labelled with the deposit symbol. However, ‘the deposit for glass bottles is only 60 öre (€0.06), which is less than for regular PET-bottles. They are also heavy, covers only certain types of glass bottles, they easily break, and you may cut yourself’ (Person 13). The transition of the DRS, from beverages being sold in refillable but heavy glass bottles to single-use cans and plastic bottles (Johansson, 2022, 2024), has thus been advantageous for the waste pickers.

It should be noted that PET-bottles with a deposit symbol, the aim of deposit pickers, constitute only about 10% of all plastic packaging sold in the Swedish market (SCB, 2023). An important reason to why no other material is collected is that waste pickers in Sweden lack access to waste buyers. Just like in other countries (Ezeah et al., 2013), the industry in Sweden only buys larger quantities of secondary materials and products from established waste companies, yet intermediaries are absent. Although individuals can sell secondary metals to scrap yards, traceability and ID checks have been implemented due to copper theft, with payments made solely to bank accounts rather than in cash. In contrast, reverse vending machines do not ask for any paperwork.

### How?

All waste pickers collect beverage containers directly from the trash bins. Just like other waste pickers, they also pick directly from the street if they see a littered deposit item. Unlike other countries (Medina, 2000; Zapata Campos et al., 2023), however, there are no organizations for waste pickers in Sweden. All interviewed deposit pickers work alone, except for two women (Person 9 and 10), who work together for the security (cf. Wittmer, 2023).

The deposit pickers use grabber tools, long-sleeved shirts and sometimes gloves, to reach deeper into the trash bins and avoid hand injuries from surrounding waste. The containers are emptied of any liquid on the sidewalk before going into the bag. Since Swedish deposit pickers focus on lightweight beverage containers and need to move quickly between sources in the urban environment, carts or shopping trolleys are rarely used, which is common for street pickers in other countries (Al-Khatib et al., 2020; Gutberlet et al., 2009; Porter, 2015). Some use bicycles for transport, but most walk and collect the beverage containers in large black garbage bags of 125 litres or smaller bags that hold about 15 litres. Like waste pickers in other countries (Zolnikov et al., 2021), many respondents report that the work is strenuous. Although each bottle and can is lightweight, the long walks each day, at a fast pace, along the diving activity into bins, result in various muscle pains, particularly in the knees and back; ‘I hurt all over, in my knees, back, and legs’ (Person 8).

After collection, the beverage containers are returned to a reverse vending machine found in most supermarkets in Stockholm. Hence, unlike traditional waste pickers, there is no need for deposit pickers to sort the collected waste. The vending machine accepts and sorts the different deposit items made of plastic or aluminium by itself, as long as the barcode is readable.

Those with smaller bags either work close to a vending machine or possess a storage. For example, one of the deposit pickers (Person 13) explained how he used a free locker at the local library as storage, which was emptied every day before the library closed.

One problem for the deposit pickers, as reported by Person 1, is that many supermarkets in Stockholm’s city centre limit the number of containers that can be returned per customer, and only offer the value to be used for shopping in the same store, meaning ‘no cashback’. These restrictions result from stores believing they receive more containers than they sell and do not want to pay dearly for extra storage space that goes to redundant waste management. This clearly shows how deposit pickers are excluded. The return system has been adapted to the store’s customers who generally return containers where they also pay the deposit, rather than the deposit pickers whose return patterns follow a different logic, based on where containers are disposed. However, the DRS operator, Returpack, has opened a separate reverse vending station in central Stockholm, without restrictions, to reduce the burden of the supermarkets.

### Why?

The vast majority (90%) of the deposit pickers state, in line with other studies in the Global North (Gutberlet et al., 2009; Rendon, 2020), that circumstances have forced them into waste picking as they lack other means of legally obtaining money. Two of these respondents also mentioned that they use the refunds to support their families in their home countries. But unlike other studies of waste pickers in the Global North (Gutberlet et al., 2009; Rendon, 2020; Rosa and Cirelli, 2018), 2 out of the 21 respondents (10%) stated that the deposit picking activity is deliberate and that they enjoy the occupation.

One of the pickers chose this occupation due to its solitary, with great autonomy; ‘With this work, I don’t need to work under anyone’ (Person 13). This sense of independence seems also to be valued by waste pickers in the Global South, as the most common form of organization is cooperatives, which offer a significant degree of autonomy in the execution of work (Buch et al., 2021; Lima et al., 2022; Medina, 2001, 2007).

The other deliberate waste picker in this study, who receives a pension, states that deposit picking is something to do and an environmental action; ‘Deposit picking is a way to occupy myself while doing a good deed for the environment. The income I get from deposits doesn’t matter to me’ (Person 7). By collecting the deposit containers from the bins, they are redirected from the incineration plant, which is the final destination of unsorted waste, to the recycling station.

**Figure 2.**
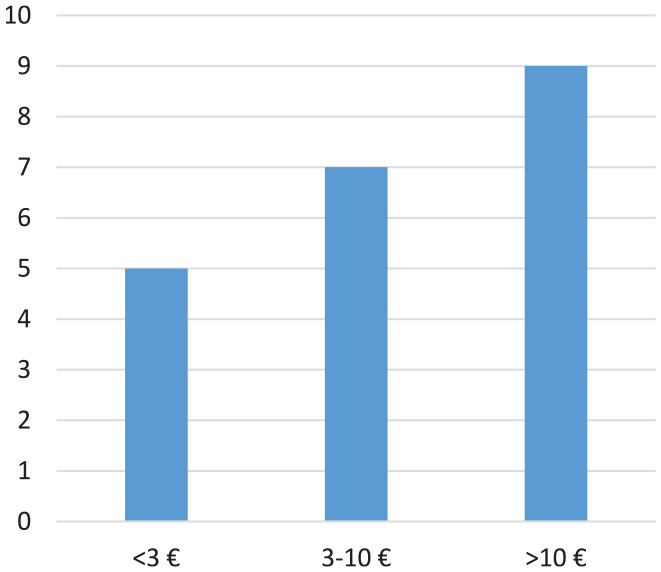
Individual earnings per day in Euros (€) and number of respondents.

Almost all (85%) respondents lack other means of support besides deposits, making the circumstances of deposit pickers more similar to waste pickers in the Global South than in the North (Medina, 2008; Rendon, 2020). In addition to the pensioner who picks for environmental benefit, one of the respondents receives income support, and a third works periodically as a carpenter during the summer to build patios, when not collecting deposits. Incomes from waste picking vary more in Sweden than in other countries (Ashenmiller, 2009; Hayami et al., 2006; Wittmer and Parizeau, 2018); ranging from 2€ to 50€ per day and person (Figure 2).

### Where?

Consumers rarely return the packaging directly after consumption. Just like other waste, consumers accumulate beverage containers at home, which are returned to the shop when the bag is full. This behaviour means that when beverages are consumed far from home, they are typically discarded in the trash bins or on the ground, since it is considered too demanding to bring them empty back home. Just like other cases have shown (Rosa and Cirelli, 2018), the wealthiest areas are thus not the most lucrative for waste pickers, but rather the places where most people pass. Therefore, the trash bins in Stockholm’s central streets and parks are prime locations for most deposit pickers.

The deposit pickers who live in the suburbs, such as person 8, usually move along their subway line, collecting bottles and cans at various stations. The subway is a typical example of a place where many people passage, consume drinks and discard containers in the public trash bins. Another popular place to collect beverage containers is at outdoor events, where people bring their own drinks. Hence, deposit pickers, like other waste pickers in the Global South (Velis et al., 2012) and North (Gutberlet et al., 2009) focus on waste in public spaces (Lane, 2011).

However, some deposit pickers employ other strategies. For example, Person 1 avoids high-traffic areas due to the increased competition. Many deposit pickers face threats and competition in these areas. One deposit picker (Person 13) reported that he was threatened with ‘a weapon’, but has now registered for a course at the university. Thereby, he obtained an access card to an area that ‘other waste pickers cannot access’, as their interests in collecting deposits were simply excluded in the planning of the area. The area is nonetheless lucrative because ‘students drink a lot’.

Another respondent (Person 6) finds recycling stations to be the most profitable and least messy places. These are usually located close to households, mostly on the street right outside, where non-beverages packaging shall be discarded. However, many households that do not return deposit cans and bottles to the reverse vending machines throw them instead into the metal and plastic containers, respectively, at the recycling stations.

### When?

**Figure 3.**
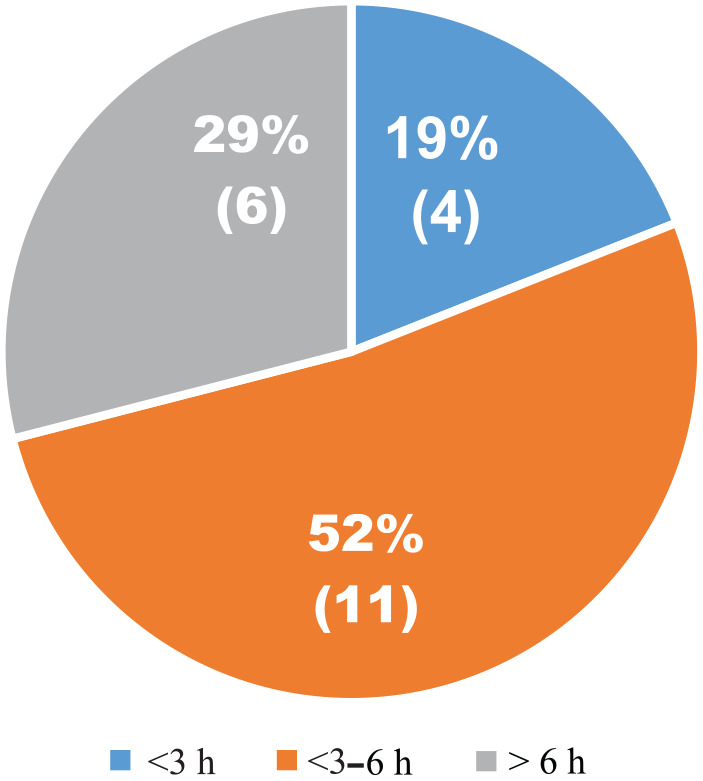
Distribution of the number of hours deposit pickers works per day. The percentages indicate the proportion of pickers, numbers in parentheses show the total pickers per category, and the colors represent the number of hours worked.

Just like waste pickers in other countries (Asim et al., 2012; Gutberlet et al., 2009; Rosa and Cirelli, 2018), deposit pickers work long hours, averaging 6 hours per day (Figure 3). However, some, like Person 8, reported that he work up to 14 hours a day.

The picking activity is relatively seasonal and relates to when people drink outdoors: ‘When it’s warm outside, I collect up to 200 bottles and cans, while in winter it’s only about 50–100’ (Person 19). During summer, when Swedes are on vacation and engage more in outdoor activities, more deposit items become available. Besides, Swedes, like other cultures, often celebrate by having a drink (Österberg and Karlsson, 2003). Holidays, school graduations or outdoor sports events are therefore times and places that are particularly interesting for deposit pickers. During winter, the deposit pickers follow people to indoor public spaces like subways, malls and train stations.

### Relationships?

The treatment of deposit pickers varies significantly. In general, Swedish-born deposit pickers experience friendliness. For example, a Swedish deposit picker in his 50s recalled that ‘A lady even wanted to give me a hug once. I pointed out that I was dirty, but she didn’t care and hugged me anyway’ (Person 13). In contrast, foreign deposit pickers, those who stated that they were born abroad, testify to poor treatment and some become emotional when it comes up. A 67-year-old deposit picker from former Yugoslavia stated, ‘I’ve been here for several years. I get no help and no medicine. Sweden and the government do nothing’ (Person 14).

This racialization of deposit pickers is also evident in the Swedish media. Swedish deposit pickers are often associated with the positive attitude of the DRS. Terms like ‘environmental hero’ (Eriksson, 2022), ‘deposit hero’ (Andersson, 2022) or ‘deposit king’ (Hörnell, 2013) are exclusively used about Swedish deposit pickers who have chosen the activity themselves. Foreign deposit pickers, on the other hand, are primarily seen as victims and have sometimes even been accused by the police and media of being associated with ‘criminals’ (Ekmark, 2015). All respondents refuted the idea that incomes from returned deposits fund criminal activities.

According to the Swedish Association of Local Authorities and Regions, trash bin owners legally own the contents. However, it is legal to pick deposit items from the trash bins because the deposit pickers ‘correct an incorrect sorting’. Nonetheless, several municipalities in the Stockholm County have completely banned the collection of deposits at recycling stations since the year 2020 (Swedish Court, 2020), which has primarily been a source for many deposit pickers from the Roma ethnic minority.

**Figure 4.**
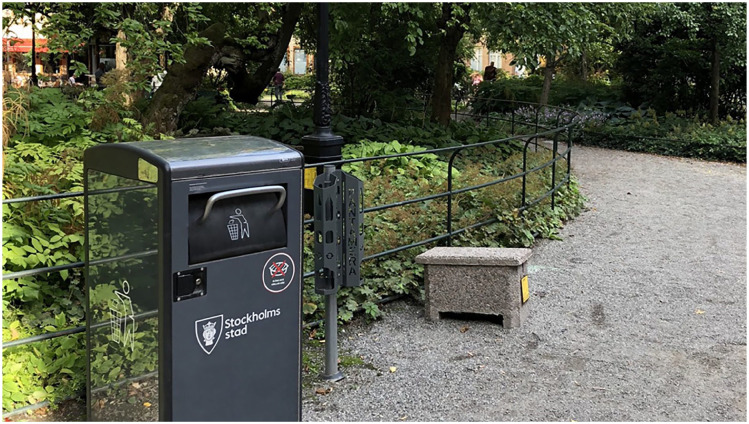
A trash bin that compresses the waste, next to deposit tubes. Note that not all compressing trash bins are equipped with deposit tubes. Used with permission by Ewfeco (2022).

Besides, those who own the trash bins report problems with littering since deposit pickers sometimes forget to close the lid of the bins. To reduce the littering and collection costs, Stockholm municipality has installed trash bins that compress the waste (Figure 4), which has obviously complicated deposit picking.

Deposit pickers are invisible in various actors’ documents, representing, for example, the DRS, anti-littering campaigns, the trash bins, recycling sector, as well as homeless people. However, Returpack, the DRS operator, responds to a direct question that deposit pickers benefit them by ‘improving collection rates and [providing] more material’ to sell. Returpack also offers municipalities to purchase so-called deposit tubes (Figure 4). These have been installed next to many trash bins and hold three to four deposit items. The intention is for people to place deposit items in these tubs, instead of in the bins, which increases the accessibility for informal pickers. However, it is unclear if the throwers actually choose to sort their deposits, as you rarely see any items stored there.

As in other parts of the world (Porras Bulla et al., 2021; Sholanke and Gutberlet, 2022), the support from the formal waste system in Sweden, exemplified through the deposit tubes or the reverse vending machines placed in public spaces, is limited to primarily benefit the system’s efficiency, rather than addressing pickers’ needs or recognizing their labour or contribution.

## Conclusion

Regardless of the waste management system and the socio-economic ranking of the country, waste picking is ubiquitous; existing in the welfare countries of the North, characterized by centralized waste systems, as well as in economically poorer countries in the Global South, where waste collection systems may be absent. The analysis of deposit pickers’ activities reveals both similarities and differences compared to traditional waste pickers, shedding light on unique but varying spatial and temporal dynamics within the context of a DRS.

A key similarity is that deposit pickers, like other waste pickers in the Global North, are predominantly older men excluded from the formal systems and thus impoverished. The primary motivation for deposit pickers and traditional waste pickers is the economic gain. The roles within the DRS are thus clearly divided by class; those who can afford, discards the deposits, which the impoverished scavenge.

A notable difference, however, is that the economic payback for deposit pickers is predetermined and does not fluctuate over time, unlike other secondary waste materials. For many respondents, deposit picking is the sole source of income, suggesting that the DRS itself may serve as a functional compensation system.

Another significant difference between the ‘planned’ DRS and the waste market, in which waste pickers traditionally operate, is that deposit pickers are compensated per collected item rather than per weight. However, in both cases, only the delivery is counted and compensated, not the pickers’ labour or their important role in correcting improper sorting, increasing recycling, reducing littering and keeping the city tidy.

DRSs are popular and effective largely thanks to the work of deposit pickers. Without them, most beverage containers consumed outside the home would be lost, and littering would increase. The benefits of deposit picking extend far beyond the deposit systems. For example, their efforts to redirect plastic bottles from incineration to recycling reduce carbon emissions.

However, since deposit pickers only collect waste marked with a deposit symbol, their societal contribution is limited compared to traditional waste pickers in the Global South, who embrace various types of waste. If more producers joined the DRS, more waste would attract deposit pickers.

On the other hand, the focus solely on beverage containers marked with a deposit symbol and the fact that the items can be deposited directly into the vending machine without any pre-sorting, lowers the threshold regarding material knowledge compared to collecting other discards. Additionally, the space and time that are most lucrative for deposit pickers differ from waste pickers. People tend to consume more beverages when they are off work, in good weather and during celebrations. During the Nordic winters, public drinking decreases, reducing the supply for pickers, who then follow consumers to indoor public spaces such as shopping malls.

The DRS also offers a single collection point for all items collected, which are more numerous and concentrated to urban areas, compared to traditional waste buyers. However, many of the reverse vending machines are located in stores that only compensate the collectors with store credits that can be used exclusively in that specific store. On the other hand, some of the reverse vending machines are unmanned, and money is paid out directly, reducing the risk of stigmatization.

The treatment from the surroundings depends on their origin of the deposit picker. Swedish pickers are celebrated as environmental heroes, while foreign pickers are portrayed as victims, sometimes associated with criminality. This stigmatization not only undermines their contribution to the recycling system but also exacerbates their social exclusion.

Mainly empty cans and PET-bottles are collected, meaning that pickers rarely need transport aids, unlike those collecting bulkier discards. Deposit pickers are often seen in long-sleeved shirts as they navigate the urban environment on foot, equipped with a sack and a grabbing tool. The low weight, ease of identification and the fact that deposit items do not need to be pre-sorted makes the activity of deposit picking more efficient than traditional waste picking. The streamlined nature of deposit picking fosters competitive relations rather than cooperation between the pickers. Organizations that unite deposit pickers are lacking in Sweden, which is available for waste pickers in many other countries.

The work and function of deposit pickers as a group are also invisible in various Swedish organizations’ governing documents, and they are generally excluded from the planning of public spaces, infrastructures and domains. If vulnerable people become dependent on the DRS, their engagement in this activity may reinforce their invisibility. Given that few of the interviewed deposit pickers received any other form of support or compensation, the deposit system seems to offer people the opportunity to live outside the formal social systems. While independence is valuable, the compensation from the DRS risks deterring vulnerably people from seeking help and engaging with the formal social assistance systems. Consequently, the DRS may displace the state’s social responsibility for this population and disrupt social interventions, support and even social reforms that addresses the root causes of people ending up in vulnerable situations.

This dual invisibility, both in terms of their lack of formal recognition and the independence from the formal social systems that comes from the income of the redeemed deposits, suggests that deposit pickers in Sweden need representation by a spokesperson. Such a spokesperson could, through cooperatives like MNCR or FACCyR in Brazil and Argentina (Kain et al., 2022), respectively, demand full recognition and compensation, not only for the collected items but also for their labour and environmental benefits. In this way, waste pickers can be integrated into formal systems and public spaces, even in the Global North. The unredeemed deposits paid annually by consumers, which today goes to the operator and corresponds to over €30 million per year, could, for example, finance such a formalization of deposit pickers. As Samson (2020) argues, it is not only the informal recyclers that should integrate with the formal waste systems, but also the formal systems need to move closer to the informal recycling systems.

## Appendix A. The interview guide

QUESTIONS FOR WASTE PICKERS:

- What type of waste do you pick?
- Where do you pick the waste?
- Why do you pick waste?
- Where were you born?
- Where do you bring the collected waste?
- How much do you earn in a day?
- How many hours do you work each day?
- What problems do you encounter through waste picking?
- How do you choose the location for waste picking?
- How do you experience the community’s response?

QUESTIONS FOR SERVICE PROVIDERS

- What is your perception of waste pickers and what are your experience with them?
- What advantages and disadvantages do they create for your operation?
- How do you manage these disadvantages and advantages?

## Appendix B. Summary of the interviewed

**Table:** Summary of the interviewed waste pickers.

Respondent reference	Gender	Age	Country of birth
1	Man	50	Sweden
2	Woman	40	Romania
3	Man	80	Sweden
4	Man	52	Sweden
5	Man	70	Sweden
6	Man	50	Romania
7	Man	65	Serbia
8	Man	56	Sweden
9	Woman	60	Sweden
10	Woman	60	Sweden
11	Man	49	Romania
12	Man	47	Latvia
13	Man	50	Sweden
14	Man	67	Former Yugoslavia
15	Man	65	Romania
16	Man	55	Sweden
17	Man	29	Bulgaria
18	Man	35	Egypt
19	Man	31	Romania
20	Man	50	Romania
21	Man	30	Burundi
